# Challenging future, challenging past: the relationship of social integration and psychological impairment in traumatized refugees

**DOI:** 10.3402/ejpt.v7.28057

**Published:** 2016-02-12

**Authors:** Matthis Schick, Andre Zumwald, Bina Knöpfli, Angela Nickerson, Richard A. Bryant, Ulrich Schnyder, Julia Müller, Naser Morina

**Affiliations:** 1 Department of Psychiatry and Psychotherapy, University Hospital Zürich, Zürich, Switzerland; 2 Faculty of Medicine, University of Zurich, Zurich, Switzerland; 3 Outpatient Clinic for Victims of Torture and War, Swiss Red Cross, Bern, Switzerland; 4 Department of Psychology, University of Bern, Bern, Switzerland; 5 School of Psychology, University of New South Wales, Sydney, NSW, Australia; 6 Psychiatric Services Thurgau, Münsterlingen, Switzerland

**Keywords:** Integration, mental health, migration, posttraumatic stress, post-migration living difficulties, PTSD, refugees

## Abstract

**Background:**

Refugees have been shown to present high prevalence rates of trauma-related mental disorders. Despite their psychological impairment, they are expected to meet high functional requirements in terms of social integration into, and financial independence from, the host society.

**Methods:**

This cross-sectional study examined the relationship of mental health problems, post-migration living difficulties (PMLD), and social integration in a sample of 104 refugees seeking treatment for severe posttraumatic stress and comorbid symptoms in two outpatient clinics in Switzerland.

**Results:**

Despite an average time of residence in Switzerland of over 10 years, participants showed poor integration and a high number of PMLD. Integration difficulties were closely associated with psychological symptoms, but not with socio-demographic parameters such as education or visa status.

**Conclusions:**

Psychological impairment in treatment-seeking traumatized refugees is associated with poor integration. To foster social integration, it is crucial to better understand and address the specific needs of this highly vulnerable population.

For the first time since World War II, the worldwide number of refugees and internally displaced persons recently exceeded 60 million (UNHCR, 2015). The experience of war and persecution referred to in the 1951 United Nations Convention Relating to the Status of Refugees implies a high risk of sustaining potentially traumatic events. Forced displacement and the often hazardous escape abroad are associated with additional threats and strains. A dose–response relationship between trauma exposure and psychological impairment is well documented (Johnson & Thompson, 2008; Mollica et al., 1998). Accordingly, refugees have generally been shown to present with high prevalence rates of mental health problems, particularly posttraumatic stress disorder (PTSD), anxiety, and depression, compared to the general western population as well as to non-refugee migrants (Fazel, Wheeler, & Danesh, 2005; Lindert, Ehrenstein, Priebe, Mielck, & Brähler, 2009; Steel et al., 2009).

Beyond the adverse effects of traumatic experiences, refugee mental health is also affected by difficulties arising *after* successful entry in a formally safe host country. Research suggests that length of asylum procedure, insecure visa status, detention in refugee camps, or prohibition to work account for a substantial proportion of psychological impairment (Brabant & Raynault, 2012; Laban, Gernaat, Komproe, Schreuders, & De Jong, 2004; Nickerson, Steel, Bryant, Brooks, & Silove, 2011; Ryan, Benson, & Dooley, 2008; Silove, Sinnerbrink, Field, Manicavasagar, & Steel, 1997). Even after eventually obtaining a secure visa status, refugees are often confronted with continuing challenges. Communication problems, financial austerity, poor accommodation, inability to find work, separation from family members, and discrimination experience have been shown to contribute to psychological distress and to the incidence of mental disorders (Laban, Gernaat, Komproe, Van der Tweel, & De Jong, 2005; Nickerson, Bryant, Brooks, et al., 2011; Nickerson, Bryant, Steel, Silove, & Brooks, 2010; Porter & Haslam, 2005).

Thus, each step of the refugee trajectory has its own characteristics and potential mental health consequences. Trauma-related factors seem to explain more variance in rates of PTSD, and post-migration appears to particularly influence rates of mood and anxiety disorders (Bogic et al., 2012). Recent research suggests, therefore, that mental health problems of refugees and asylum seekers are best captured by models integrating pre- and post-migration factors (Miller & Rasmussen, 2010; Nickerson, Bryant, Silove, & Steel, 2011).

After obtaining residence authorization, refugees are usually expected, or legally obliged, to rapidly participate in the host society, particularly regarding language proficiency and financial independence. The process of social integration implies high functional requirements in terms of cognitive and interpersonal capabilities, which refugees with psychological impairments are often not able to meet. The relation between mental health problems and integration difficulties of refugees has attracted little research attention. In a large-scale dataset including about 4,000 refugees, post-migration stressors were found not only to affect mental health but also to hinder socioeconomic integration (Bakker, Dagevos, & Engbersen, 2014). Another study conducted with 3,000 refugees concluded that general health problems and depression were negatively associated with successful economic integration (De Vroome & Van Tubergen, 2010). While policies that aim to foster language proficiency and labor market participation are increasingly established in order to assist healthy refugees in their process of integration, little is known about needs and limitations regarding the integration of the large proportion of refugees with psychological impairments. Questions such as how to screen for, how to best support, and what to expect from mentally ill refugees regarding their integration are of crucial importance for policymakers. Therefore, comprehensive research that addresses gaps, methodological challenges, and eventually preventive interventions is strongly recommended (Abebe, Lien, & Hjelde, 2014).

The present cross-sectional study aimed to contribute to knowledge about the relationship of social integration and mental health problems in a sample of refugees seeking treatment for severe posttraumatic stress and comorbid symptoms in two outpatient clinics for victims of torture and war in Switzerland. Based on sound evidence on functional impairment related to PTSD and depression, we hypothesized that successful social integration would be negatively related to symptom scores of PTSD and depression (e.g., Momartin, Silove, Manicavasagar, & Steel, 2004; Olff, Polak, Witteveen, & Denys, 2014). Based on the limited literature on predictors of economic integration of refugees, we further hypothesized that successful social integration would be related to potentially favorable socio-demographic factors such as higher education and secure visa status (for overview see De Vroome & Van Tubergen, 2010; Fozdar & Hartley, 2013).

## Methods

### Participants

Participants were in treatment in two outpatient units for victims of torture and war in Switzerland. Due to the cross-sectional study design, participants were in different stages of therapy, including pretreatment, treatment, and posttreatment stages. Treatment included trauma-specific as well as unspecific psychotherapy, medication, and social counseling, depending on symptom profiles and subjective focus of distress. Patients aged 18 years or older and speaking one of the study languages (German, English, Turkish, Arabic, Farsi, or Tamil) were included in the study. Asylum seekers were not included, as Swiss law does not encourage social integration prior to completion of the asylum procedure, and therefore, a reliable conclusion on integration capacity would not have been possible for structural reasons. Exclusion criteria included current psychotic symptoms, severe dissociative symptoms, and acute suicidality. Of the 122 patients that were invited, 107 patients (87.7%) agreed to participate in the study. Of these, three patients failed to attend the research session. Finally, *N=*104 participants were assessed.

### Measures

All measures used in this study were translated and back-translated by accredited translators in accordance with gold-standard translation practices (Bontempo, 1993). Discrepancies were rectified jointly by the research team and independent bilingual individuals who were experienced in working with health-related questionnaires.

Exposure to traumatic events was indexed using a measure derived from combining the trauma event lists of the Harvard Trauma Questionnaire (HTQ; Mollica et al., 1992) and the Posttraumatic Diagnostic Scale (PDS; Foa, 1996; Foa, Cashman, Jaycox, & Perry, 1997). Overall trauma exposure was represented by a count of the number of traumatic event types experienced by each participant.

Symptoms of PTSD in the past month were measured using the PDS, with four additional items being included consistent with the DSM-5 criteria for this disorder (American Psychiatric Association, 2013). The scale yields a continuous PTSD symptom score and has been used with numerous refugee groups (e.g., Neuner, Schauer, Klaschik, Karunakara, & Elbert, 2004; Norris & Aroian, 2008). Cronbach's alpha in this study was α=.94.

Depression and anxiety in the last week were measured using the respective subscales of the Hopkins Symptom Checklist (HSCL-25; Mollica, Wyshak, de Marneffe, Khuon, & Lavelle, 1987). This scale has been employed with a number of refugee groups (e.g., Carlsson, Mortensen, & Kastrup, 2005; Schweitzer, Melville, Steel, & Lacherez, 2006). We computed a total continuous score of depression (range=1–4) and anxiety (range=1–4) symptoms. Cronbach's alpha was α=.91 for anxiety and .90 for depression.

Health-related quality of life and functional impairment were measured using the physical and mental component score of the 12-item Medical Outcomes Study-Short Form (SF-12; Gandek et al., 1998; Ware, Kosinski, & Keller, 1996). Psychometric properties and factor structure of the SF-12 have been examined in several studies worldwide (e.g., Montazeri et al., 2011).

Social integration was assessed according to 11 key domains upon which the Swiss government based its national monitoring of social integration of the immigrant population (Swiss Federal Office of Statistics, 2015). As no established instrument existed for these indicators, we used a version of the Post-Migration Living Difficulties Checklist (PMLDC; Silove et al., 1997; Steel, Silove, Bird, McGorry, & Mohan, 1999) adapted to the Swiss context. This 17-item scale (range = 0–68) has been successfully used in previous studies (Nickerson, Bryant, et al., 2015; Nickerson, Schnyder, et al., 2015) and examined the extent to which post-migration challenges had been of concern over the past 12 months. Items are rated on a five-point scale (0 = not a problem to 4 = very serious problem). Items scored at least 2 (moderately serious problem) were considered positive responses, yielding a total count of living difficulties. This scale has consistently been identified as a predictor of mental health among displaced populations (Nickerson et al., 2010; Schweitzer et al., 2006; Steel et al., 2006). Seven items of the PMLDC were found to correspond to the respective key domains of integration and were subsequently used to describe integration difficulties. The items encompassed language (“communication difficulties”), employment (“difficulties with work”), access to health care (“worries not receiving medical treatment”), financial situation (“not enough money for food, clothes, or rent”), accommodation (“difficulties finding appropriate housing”), social participation (“isolation, boredom, and loneliness”), and discrimination. Four of the 11 domains were not covered by the assessment: criminality rate, political participation, post-migration education, and family characteristics such as rates of working mothers or child care utilization. A continuous score was used to measure difficulties with integration. The resulting subscale (range=0–28) showed reasonable internal consistency with Cronbach's alpha α=.72. The other PMLDC items were included in the assessment of PMLD, but not of integration difficulties, as they were predominantly attributed to structural limitations, for example, visa status, instead of limitations in integration capacity.

### Procedure

The study was approved by the Ethics Committees of the Cantons of Zurich and Bern. Written informed consent was obtained, with participants being informed they were free to withdraw from the study without influence on future treatment. Questionnaires were applied using a therapist-assisted computer-based assessment tool (MultiCASI, Knaevelsrud & Müller, 2008). In MultiCASI, self-report questionnaires are presented on an electronic tablet in written and auditory form in the respondent's mother tongue. Assessments were supervised by a clinical psychologist or a masters-level student of clinical psychology. Participants were reimbursed CHF 40 (approx. USD 40) for participation.

### Data analysis

Analyses were conducted using SPSS Version 22. There was less than 5% missing data on any of the variables included in the analyses. Descriptive statistics are given in terms of means and standard deviations in continuous variables, and counts and percentages in categorical variables. The relationship between psychopathological symptoms and social integration was examined by means of Pearson's correlations. As psychological parameters and health-related quality of life were highly interdependent, multiple regression analysis including all of these factors produced no meaningful results. Separate hierarchical linear regression analyses (method: enter) were conducted for PTSD severity and depression, respectively, with integration difficulties as the outcome variable. For each model, age, trauma count, and length of stay in Switzerland were entered at the first step. PTSD or depression scores were then entered at the second step to examine the extent to which it predicted integration difficulties.

## Results

Sample characteristics are shown in Table 1. Participants were, on average, 43 years old (SD=9.3); 79% were male, in line with the gender distribution among asylum seekers in Switzerland; and over 60% had 8 or more years of education. Despite a length of residence in Switzerland of over 10 years (SD=6.7), social integration in general was remarkably poor: The mean integration difficulties score was *M*=20.7 (SD=6.1, scale range=0–28), corresponding to serious integration difficulties. Difficulties with isolation, employment, and communication were experienced most commonly. Only 22% of the sample had a full-time or part-time employment, and 86% were financially dependent on others, mostly on social welfare. Only 18% of the sample had sufficient language proficiency to answer the questionnaires in German. Participants reported a mean of *M*=9.7 (SD=4.2, scale range=0–17) types of living difficulties as representing a moderately serious, serious, or very serious problem (see Table 2). In addition to the mentioned integration difficulties, worries related to family members left behind were of particular concern.

**Table 1:** Sample description (*N*=104)

Characteristics	*n*	%
Male gender	82	78.8
Ethnicity		
Turkey	58	55.8
Middle East	22	21.1
Sri Lanka	8	7.7
Former Yugoslavia	8	7.7
Other	8	7.7
Marital status		
Married/relationship	65	62.5
Divorced/widowed	14	13.5
Single	25	24.0
Education, years		
Less than 4	17	16.3
4–8	21	20.2
8–12	29	27.9
12 +	35	33.7
Employment		
Employed full-/part-time	23	22.1
Unemployed	58	55.8
Retired/homemaker	20	19.2
Financial support		
Employment	15	14.4
Family members	8	7.7
Social welfare	68	65.4
Other	11	10.6
Visa status		
Temporary	13	12.5
Permanent	76	73.1
Citizenship	15	14.4
Age, years (SD)	43.4	(9.34)
Stay in Switzerland, years (SD)	10.6	(6.71)
Mean number of PTE (SD)	12.6	(4.3)

PTE, potentially traumatic event types.

**Table 2:** Post-migration living difficulties (PMLD) reported as moderately serious, serious, or very serious problem (past year)

PMLD type	*n*	(%)
*Loneliness, boredom, or isolation*	88	84.3
Worries about family back home	83	80.6
Being unable to return to your home country in an emergency	81	79.4
Difficulty learning German	78	75.0
Separation from family	75	72.1
*Difficulties with employment*	71	69.6
*Communication difficulties*	65	63.1
Being fearful of being sent back to your country of origin	59	56.7
Difficulties obtaining financial assistance	59	56.7
*Difficulty obtaining appropriate accommodation*	55	52.9
*Not enough money for food, rent, or necessary clothes*	54	52.4
*Discrimination*	51	49.0
*Worries about not getting treatment for health problems*	46	44.7
Conflicts with social workers/other authorities	41	39.4
Difficulties in interviews with immigration officials	38	36.5
Not being recognized as a refugee	35	33.7
Ethnic conflicts	26	25.5
Mean number (SD) of PMLD per participant	9.7	(4.2)

Items included in the Integration Difficulties Subscale are given in italics.

With regard to traumatic experiences, participants reported a mean of *M*=12.6 (SD=4.3) types of potentially traumatic event types. Roughly 87% of the participants had experienced torture, 80% had experienced combat situations, and 74% reported having been close to death. Accordingly, the mean PTSD symptom scores were *M*=1.7 (SD=.67), corresponding to a moderate to severe symptom severity. The large standard deviation indicates that there is substantial variability in PTSD scores, which may reflect the fact that participants were in different stages of therapy. Symptom scores were *M*=2.7 for depression (SD=.62) and *M*=2.7 for anxiety (SD=.69), respectively, with scores exceeding 1.75 suggesting probable diagnosis. The latency between entering Switzerland and admittance to therapy was *M*=7.7 years (SD = 6.57) on average, suggesting a predominance of highly chronic illness.

The results of the correlation analyses are shown in Table 3. Contrary to our expectations, no correlations were found between integration and socio-demographic parameters (i.e., gender, education, or visa status). As predicted, however, difficulties in social integration correlated significantly with health-related quality of life and functional impairment (*r*=−.47), symptom severity of depression (*r*=.44), PTSD (*r*=.43), and anxiety (*r*=.29). As anxiety was highly interrelated with PTSD and depression and demonstrated a relatively weaker relationship with social integration, it was not included in the regression analysis. In accordance with our hypothesis, regression analysis revealed that after controlling for the effects of age, traumatic experience, and time in Switzerland, symptoms of both PTSD and depression predicted difficulties in integration (see Table 4).

**Table 3:** Pearson's correlations of difficulties in social integration with socio-demographic characteristics, traumatic experiences, psychopathology, health-related quality of life and functional impairment (*N=*104)

	Integration difficulties	Gender	Age	Education	Visa status	Stay in CH	Trauma	PTSD	Depression	Anxiety
Gender	−.021									
Age	−.196*	−.074								
Education	.069	−.087	.089							
Visa status	−.193	−.065	.328**	−.113						
Stay in CH^a^	−.315**	−.062	.506***	−.127	.521***					
Trauma^b^	.259**	−.175	−.090	.163	−.088	−.078				
PTSD^c^	.433***	−.030	−.029	−.022	.073	.068	.280**			
Depression^d^	.441***	.030	−.022	−.028	.025	.050	.245*	.772***		
Anxiety^e^	.293**	.119	−.031	−.032	−.004	.017	.240*	.639***	.772***	
SF-12^f^	−.465***	−.050	−.022	.075	−.058	−.129	−.238*	−.677***	−.682***	−.521***

a CH: Switzerland;

b Harvard Trauma Questionnaire, Posttraumatic Diagnostic Scale;

c Posttraumatic Diagnostic Scale;

d Hopkins Symptom Checklist 25, Subscale Depression;

e Hopkins Symptom Checklist 25, Subscale Anxiety;

f 12-item Medical Outcomes Study-Short Form.

* *p*<.05,

** *p*<.01,

*** *p*<.001.

**Table 4:** Hierarchical linear regression analyses of integration difficulties, PTSD, and depression

Outcome variable	Independent variable		*B*	SEB	*β*	*T*	*p*	*F*	*p*	*R* ^*2*^	*R* ^*2*^ adjusted
Integration difficulties	Step 1	Age	−.003	.063	−.005	−.050	.960	11.578	.000	.319	.291
		Trauma count	.165	.125	.114	1.315	.192				
		Length of residence	−.304	.089	−.332	−3.431	.001				
	Step 2	PTSD^a^	3.872	.795	.424	4.871	.000				
	Step 1	Age	−.009	.063	−.013	−.139	.890	11.868	.000	.324	.297
		Trauma count	.186	.123	.129	1.506	.135				
		Length of residence	−.292	.088	−.319	−3.319	.001				
	Step 2	Depression^b^	4.194	.844	.425	4.971	.000				

a Posttraumatic Diagnostic Scale;

b Hopkins Symptom Checklist 25, Subscale Depression.

## Discussion

This cross-sectional study examined the relationship between social integration and mental health problems in a clinical sample of 104 refugees undergoing treatment in two outpatient clinics for victims of torture and war in Switzerland. Participants reported high levels of exposure to potentially traumatic experiences and were suffering from substantial psychological impairment. Numerous earlier studies have demonstrated the detrimental long-term effects of severe and multiple traumatic experiences not only in clinical samples, but in refugee populations in general (Marshall, Schell, Elliott, Berthold, & Chun, 2005; Mollica et al., 2001; Steel et al., 2009; Steel, Silove, Phan, & Bauman, 2002).

Despite comparably high education and long duration of residency in Switzerland, participants in general showed remarkably poor integration, particularly in terms of labor market participation and language proficiency, and were subject to a high number of migration-associated living difficulties. These findings are in line with many studies on traumatized refugees demonstrating the relationship of ongoing psychological impairment and overextending post-migration living conditions, even if formal safety is achieved (Bogic et al., 2012; Laban et al., 2005; Silove et al., 1997). Contrary to our expectations, potentially advantageous socio-demographic factors such as high education and secure visa status did not translate into lower levels of reported integration difficulties. Integration difficulties, however, were strongly associated with symptom scores of PTSD and depression, over and above the number of traumatic events to which the individual had been exposed. These findings suggest that, in a clinical sample, the adverse effects of psychological impairment may overshadow the potentially beneficial effects of socio-demographic resources in terms of successful integration.

The association of psychological symptoms with social and functional impairment is perhaps unsurprising as impairment informs the diagnostic criteria of many DSM-5 disorders (“… causes […] impairment in the individual's social interactions, capacity to work or other important areas of functioning,” American Psychiatric Association, 2013). With regard to the integration of traumatized refugees, however, our results show that existing approaches and policies may not be sufficiently effective. Under the premise that host societies intend for refugees to successfully integrate (particularly in terms of their financial independence), our findings suggest several issues as being critically important.

A first critical issue is the availability and utilization of adequate health care. Although Switzerland is providing refugees and asylum seekers with mandatory health insurance, participants of our study had to suffer for almost 8 years on average before they were referred to a specialized mental health service. Although evidence-based treatment options for PTSD and other psychiatric disorders exist (e.g., Schauer, Neuner, & Elbert, 2005; Schulz, Resick, Huber, & Griffin, 2006), the timely access to mental health care for refugees and asylum seekers is often hindered by language barriers, distrust, stigma, and/or the lack of knowledge about psychological disorders and treatment possibilities (Morris, Popper, Rodwell, Brodine, & Brouwer, 2009). In a Dutch study, over 90% of asylum seekers with a psychiatric disorder did not visit a mental health service in the 2 months prior to assessment (Laban, Gernaat, Komproe, & De Jong, 2007). A longitudinal study on refugees resettled in the Netherlands showed that only 21% of respondents with PTSD had had contact with a mental health care provider at baseline assessment, and after a 7-year follow-up, the overall prevalence of PTSD remained unchanged (Lamkaddem et al., 2014).

However, challenges arise not only in terms of access to and utilization of health care, but also in relation to health care providers. In a Swiss study, mentally ill asylum seekers were often underdiagnosed and inadequately treated compared to Swiss residents (Maier, Schmidt, & Mueller, 2010). A study assessing the quality of primary health care for resettled refugees with chronic mental and non-communicable health problems in the Netherlands revealed that only 50% of common mental disorders were recognized by the general practitioner (Van Melle et al., 2014). Though many governments pursue the goal of enabling all citizens to have equal opportunities to access health care, the needs of refugees in terms of access to appropriate treatment are often not met. Given the high incidence of refugees with mental health problems and the possible negative implications for future integration, our findings highlight the crucial importance of early identification and adequate treatment of refugees in need.

A second important issue emerging from our findings concerns the vigorously debated question regarding what should comprise best practice treatment of traumatized refugees with mental health problems. While there is agreement concerning the mental health impact of posttraumatic and post-migration stressors, two opposing approaches are promoted. Advocates of trauma-focused interventions argue that an improvement of PTSD symptoms will lead to an improvement in functionality and better adaption to impending challenges of resettlement and acculturation. In contrast, exponents of multimodal interventions argue that an exclusive focus on exposure techniques may not be sufficient or even result in adverse emotional reactions. Instead, they suggest a range of psychosocial interventions for the purpose of general stabilization, which, in turn, would allow better management of traumatic stress symptoms. While the literature provides evidence for the effectiveness of trauma-focused treatment in reducing symptoms of PTSD among refugees, this does not hold for approaches exclusively focusing on counseling or multimodal interventions. However, the long-term effects of these interventions on other mental health problems or on functional impairment have not been assessed in these studies (for review see Nickerson, Bryant, Silove, et al., 2011). Furthermore, post-migration stress factors such as unemployment and family separation were found to predict the maintenance of PTSD symptoms in resettled refugees (Lie, 2002). In addition, increased PMLD were negatively related to an improvement in PTSD (Lamkaddem et al., 2014). Numerous environmental factors (e.g., temporary residence permit or long asylum procedures) have been shown to be independently associated with higher rates of mood and anxiety disorders (Bogic et al., 2012). Given the close relationship between social and psychological factors found in our study, we suggest that models and treatment of refugee mental health need to recognize the wide variety of problems that may be experienced by traumatized refugees (Patel, Kellezi, & Williams, 2014). Therapists may benefit from implementing interventions that target those symptoms causing the greatest distress, and working with the client to balance the potential benefits of alleviating trauma-related and psychosocial distress in a way that optimally facilitates functional improvement (Nickerson, Bryant, Silove, et al., 2011).

A third critical point in terms of adequate mental health care for refugees and asylum seekers is communication. Despite having lived in Switzerland for over 10 years on average, less than 20% of the participants in our study had been able to acquire sufficient language proficiency to answer the questionnaires in German. A monitoring report on asylum seekers and refugees from Somalia and Sri Lanka commissioned by the Swiss Federal Office of Public Health came to the conclusion that only little more than one-third of the Somali and one-fourth of the Tamil refugees were able to explain, unassisted, their concerns to the doctor, or to understand their doctor's questions and instructions correctly (Guggisberg et al., 2011). Language barriers between doctors and patients have been associated with lower health education, lower quality of care, higher rates of adverse events, longer length of stay in hospital and higher readmission rates (Divi, Koss, Schmaltz, & Loeb, 2007; Flores, 2005; Karliner, Jacobs, Chen, & Mutha, 2007; Lindholm, Hargraves, Ferguson, & Reed, 2012; Ngo-Metzger et al., 2007; Ribera, Hausmann-Muela, Grietens, & Toomer, 2008). Although the use of qualified medical interpreters is advised (Crosby, 2013), the limiting factor is often additional cost. Though usual practice in legal proceedings, the use of qualified interpreters is unfortunately far from being a standard in the medical setting.

### Limitations

A major limitation of the current study concerns the assessment of social integration. Due to the complexity and the indistinct definition of the theoretical construct, no appropriate and established instruments were available. We therefore approximated the respective indicators of social integration provided by the Swiss government as closely as possible by adapting the widely used PMLD Checklist so that essential dimensions of integration were covered by our assessment. Second, while we used transculturally validated measures whenever available, participants were from numerous cultural backgrounds, and thus, it was not possible to use measures validated with each cultural group. Third, self-report measures were implemented instead of clinician administered diagnostic interviews. A fourth limitation is the cross-sectional design that precludes inferences about causality. In addition, participants were assessed in different stages of therapy. Though chronic illness was highly predominant in our sample, we cannot rule out an incongruity between short-term treatment effects on symptom scores and integration difficulties, which are typically subject to a long-term process. Finally, this study assessed a clinical sample of treatment-seeking refugees, and therefore, the results cannot be extended to traumatized refugees in general.

## Conclusions

Though longitudinal studies are needed to explore causal relations, our findings contribute to the comprehension of a circular association between psychiatric illness related to posttraumatic and post-migration stress, functional impairment, and PMLD. Refugees with high levels of psychiatric comorbidity and psychosocial impairment may have difficulties in regulating the ongoing effects of stress on their functional capacity, and the effects of post-migration difficulties contribute to ongoing mental health problems. This is particularly relevant to survivors of extreme interpersonal trauma who by virtue of their preexisting mental health conditions and other related factors (e.g., mental health literacy, stigma, and suspicion of authorities) may be less likely to seek help. The close association of integration difficulties and psychological symptoms found in our sample suggests that efficacious and timely treatment of mental health conditions could possibly enhance successful integration of traumatized refugees in the host society. Considering the increasing numbers of refugees across the world, and the significant personal and societal costs associated with their resettlement, it is imperative to develop more effective interventions to facilitate optimal adjustment for refugees with impaired mental health.
